# Dysregulated autophagic activity induced in response to chronic intermittent hypoxia contributes to the pathogenesis of NAFLD

**DOI:** 10.3389/fphys.2022.941706

**Published:** 2022-08-02

**Authors:** Dong Wang, Dongyu Si, Gang Li, Zhimin Ding, Xiaonan Yang, Chaobing Gao

**Affiliations:** ^1^ Department of Otorhinolaryngology Head and Neck Surgery, First Affiliated Hospital of Anhui Medical University, Hefei, China; ^2^ Department of Otorhinolaryngology Head and Neck Surgery, Fuyang Hospital of Anhui Medical University, Fuyang, China

**Keywords:** osa, chronic intermittent hypoxia, autophagy, endoplasmic reticulum stress, NAFLD

## Abstract

Chronic intermittent hypoxia (CIH) is a pathological characteristic of obstructive sleep apnea (OSA) that has been linked to the pathogenesis of nonalcoholic fatty liver disease (NAFLD). The specific link between CIH, autophagic activity, and NAFLD, however, has not previously been characterized. The goal of this study was to assess the relationship between CIH-induced autophagy and the pathogenesis of OSA-associated NAFLD. Western blotting was used to assess the expression of proteins associated with lipid synthesis, endoplasmic reticulum (ER) stress, and autophagic activity. To establish an *in vivo* model system, C57BL/6 mice were subjected to CIH conditions for 8 h per day over a 12-week period, and were administered chloroquine (CQ) for 1 week prior to euthanization. Levels of serum and liver triglycerides in these animals were assessed, as were proteins related to hepatic autophagy, ER stress, and lipogenesis. qPCR was additionally used to assess hepatic inflammation-related gene expression, while transmission electron microscopy was used to monitor lipid droplet (LD) accumulation and ER morphology. OSA patients and CIH model mice exhibited increases in the expression of proteins associated with hepatic autophagy, ER stress, and lipogenesis. CIH was also associated with more pronounced LD accumulation, hepatic inflammation, and hepatic steatosis in these mice. While serum and hepatic TG and TC levels and serum ALT/AST were increased in response to CIH treatment, the administration of CQ to these mice led to reductions in ER stress-related proteins (XBP1, IRE1α, EIF2α) and lipogenesis-related proteins (ACC, SCD1, FASn), in addition to significantly reducing hepatic inflammation, steatosis, and LD accumulation in these animals. These results suggest that persistent CIH can drive dysregulated hepatic autophagic activity, hepatic steatosis, and ER stress, highlighting potential targets for therapeutic intervention aimed at preventing or treating OSA-associated NAFLD.

## Introduction

Obstructive sleep apnea (OSA) is a common sleep disorder in which patients experience the partial or total collapse of the airway while sleeping, resulting in chronic intermittent hypoxia (CIH) that can, in turn, contribute to low-level oxidative stress and inflammatory activity (Arnaud et al., 2009). A number of adverse metabolic conditions have been linked to OSA, including insulin resistance and metabolic syndrome (Tasali and Ip, 2008; Martinez-Ceron et al., 2016; Javaheri et al., 2017)**.** CIH is a key facet of the pathogenesis of OSA that has been specifically correlated with metabolic syndrome (Musso et al., 2013; Borel, 2019; Kuvat et al., 2020), and the primary hepatic consequence of metabolic syndrome is the development of nonalcoholic fatty liver disease (NAFLD) (Kim and Younossi, 2008). As the most prevalent form of chronic liver disease, NAFLD patients exhibit steatosis associated with the accumulation of lipid droplets (LDs) within hepatocytes, ultimately resulting in progression to nonalcoholic steatohepatitis (NASH), fibrosis, and an elevated risk of liver cancer and cirrhosis (Dulai et al., 2017). While many prior studies have identified risk factors tied to the development of NAFLD, it remains a highly heterogeneous disease, and the molecular mechanisms governing its progression remain to be fully clarified. In clinical studies, OSA has been found to be related to NAFLD onset and progression independent of obesity or other relevant risk factors (Mesarwi et al., 2019). While continuous positive airway pressure (CPAP) treatment is the first-line therapeutic option for OSA patients and can alleviate nocturnal CIH, one recent randomized clinical trial found the CPAP treatment of OSA patients with NAFLD to have no significant impact on triglyceride (TG) levels within the liver (Ng et al., 2021). As such, further research is needed to elucidate the complex pathological mechanisms linking OSA and the pathogenesis of NAFLD.

Animal model research focused on OSA to date has primarily explored the relationship between CIH and NAFLD progression mediated by hypoxia-inducible factor (HIF) (Chen et al., 2019; Holzner and Murray, 2021; Mesarwi et al., 2021). When subjected to CIH treatment, cells engage stress responses aimed at restoring homeostatic conditions, with the induction of autophagy being one key component of these stress responses. Notably, autophagy has also been linked to the pathogenesis of NAFLD, the inhibition of autophagy has been reported to contribute to TG storage within LDs, thereby driving LD accumulation and associated hepatic steatosis (Yang et al., 2010). In mouse model systems, pharmacological manipulation of the autophagy pathway can alleviate NAFLD and NASH(Lin et al., 2013), while the knockout or inhibition of autophagy-associated genes has similarly been reported to suppress LD accumulation and to thereby protect against hepatic steatosis (Nguyen et al., 2017; Li et al., 2018). Despite some uncertainty regarding the precise role that autophagy plays in the context of NAFLD, it is thus nonetheless an important regulator of hepatic steatosis development.

Recent evidence suggests that endoplasmic reticulum (ER) stress can profoundly contribute to the pathogenesis of NAFLD (Lebeaupin et al., 2018), with some studies having reported a link between ER stress, autophagic activity, and NAFLD development (Flessa et al., 2021). The disruption of normal autophagic activity within the liver can promote ER stress induction and the onset of insulin resistance in obese individuals (Yang et al., 2010). Acute intermittent hypoxia has been reported to protect against ER stress and apoptotic death via adaptive increases in cardiac autophagy (Chang et al., 2019), yet it remains unclear as to whether CIH can induce dysregulated autophagic activity in hepatocytes that leads to ER stress.

In the present study, ER stress- and autophagy-associated protein levels were found to be elevated in hepatocytes from individuals diagnosed with OSA relative to control hepatocytes. Further analyses were then conducted with the aim of determining the role played by autophagy and ER stress in the pathogenesis of CIH-induced hepatic steatosis.

## Materials and methods

### Patient samples

Hepatic samples were collected from patients underwent bariatric surgery or liver tumor underwent hepatic resection at our institution that were or were not diagnosed with OSA, as diagnosed via preoperative polysomnography (PSG), prior to surgery. Patients diagnosed with normal or NAFLD via post-operative liver pathologic results. All participants received a clinical examination and without any history of liver diseases. All samples were stored at −80°C, and the Institutional Review Board of the First Affiliated Hospital of Anhui Medical University approved all studies.

### Animal model establishment

Male 5-week-old C57BL/6 mice were obtained from the Model Animal Research Center (Nanjing, China). After acclimatization for 1 week during which mice were fed a basal diet in the Laboratory Animal Center of Anhui Medical University, mice were randomized into three groups (*n* = 4/group): a control group, a CIH group (CIH exposure for 12 weeks), and a CIH + CQ group [CIH exposure for 12 weeks, within intraperitoneal chloroquine (CQ, 60 mg/kg/d) for the last 1 week of the treatment period (Lin et al., 2013)].

To establish a model of CIH, all mice were assigned to special chamber that were equipped with a computer-controlled gas delivery system that provided alternating cycles of oxygen and nitrogen. Fractional inspired O_2_ (FiO_2_) levels were decreased from 21 ± 1% to 6 ± 1% within 40 s, remained at that concentration for 20 s, and then returned to 21 ± 1% within the next 40 s, remaining at that concentration for an additional 20 s. This CIH exposure was maintained each day from 08:00 to 16:00 daily for a 12-week exposure period.

### Analyses of serum glucose, serum and hepatic lipid levels

Serum alanine aminotransferase (ALT), aspartate aminotransferase (AST), glucose, TG and total cholesterol (TC) levels were assessed with an automated analyzer (Roche Modular DPP). TG and TC levels in liver samples were assessed with a colorimetric diagnostic kit (Applygen Technologies, Inc, Beijing, China) based on provided directions. Final TG and TC concentrations were adjusted based on protein content levels.

### Histological and transmission electron microscopy analyses

After fixation with 10% neutral-buffered formalin, liver sections were paraffin-embedded, cut into 5 µM sections, and subjected to hematoxylin and eosin (H&E) staining. Oil Red O staining was performed by embedding samples using an OCT compound, cutting them to a thickness of 30 µM, and staining with Oil Red O to facilitate neutral lipid identification (red), with hematoxylin (blue) used for nuclear counterstaining. TEM was performed based on a previously published protocol (Wu et al., 2016).

### Quantitative real-time PCR

TRIzol (Invitrogen) was used to isolate total cellular and tissue sample RNA, after which cDNA was prepared from 1 ug of RNA per sample with a reverse transcription kit (Thermo). Then, qPCR was conducted with a Bio-Rad CFX system and a SYBR Green I qPCR kit (Promega). The 36B4 reference gene was used to normalize all gene expression data. Primers sequences were synthesized by Tsingke Biotechnology Co., Ltd and are showed in Table 1.

**TABLE 1:** Primers sequences for quantitative real-time PCR.

Target gene	Forward primer sequences	Reverse primer sequences
TNF-α	5′-AAT​GGC​CTC​CCT​CTC​ATC​AGT​T-3′	5′-CCA​CTT​GGT​GGT​TTG​CTA​CGA-3′
IL-6	5′-TAG​TCC​TTC​CTA​CCC​CAA​TTT​CC-3′	5′-TTG​GTC​CTT​AGC​CAC​TCC​TTC-3′
IL-1β	5′-GAA​ATG​CCA​CCT​TTT​GAC​AGT​G-3′	5′-TGG​ATG​CTC​TCA​TCA​GGA​CAG-3′
FASn	5′-GTA​AGT​TCT​GTG​GCT​CCA​GAG-3′	5′-GCC​CTC​CCG​TAC​ACT​CAC​TC-3′
SCD-1	5′-CTG​CAC​CTC​CCT​CCG​GAA​AT-3′	5′-TCC​TCC​AGA​CGT​ACT​CCA​GC-3′
ACC	5′-AGG​AAG​ATG​GCG​TCC​GCT​CTG-3′	5′-GGT​GAG​ATG​TGC​TGG​GTC​AT-3′

### Western blotting

RIPA buffer (Cat.No.P0013B, Beyotime, China) was used to lyse liver tissue samples, after which extracted proteins were separated via 12% SDS-PAGE and transferred onto PVDF membranes (Millipore, MA, United States). Blots were blocked using 5% non-fat milk in TBST for 1 h, followed by overnight incubation at 4°C using a anti-EIF2α (1:1,000, Cat.No.5324S, CST, United States), anti-p-EIF2α (1:1,000, Cat.No.3398T, CST, United States), anti-IRE1α (1:1,000, Cat.No.3294S, CST, United States), anti-XBP1 (1:1,000, Cat.No.PB9463, Boster, China), anti-LC3A/B (1:1,000, Cat.No.12741S, CST, United States), anti-p62 (1:1,000, Cat.No.23214S, CST, United States), anti-Beclin1 (1:1,000, Cat.No.3459T, CST, United States), anti-ACC (1:1,000, Cat.No.21923-1-AP, Proteintech, China), anti-SCD1 (1:1,000, Cat.No. SC-515844, Santa Cruz,United States), anti-FASn (1:1,000, Cat.No. SC-48357, Santa Cruz, United States), anti-β-Actin (1:1,000, Cat.No.4970S, CST, United States). Blots were then incubated with appropriate HRP-conjugated secondary antibodies for 1 h with constant shaking at room temperature, after which they were washed four times in 1x TBST. Protein bands were then detected with a chemiluminescence apparatus via an ECL approach. All bands were analyzed semi-quantitatively using ImageJ software.

### Statistical analysis

Data were analyzed using GraphPad Prism 9. Comparisons between two groups were subjected to Student’s t test. Comparisons between more than two groups were subjected to one-way ANOV A test followed by Tukey’s post hoc test. *p* < 0.05 was the threshold of statistical significance.

## Results

### Obstructive Sleep Apnea patients exhibit elevated hepatic levels of proteins associated with endoplasmic reticulum stress, autophagy, and steatosis

**FIGURE 1:**
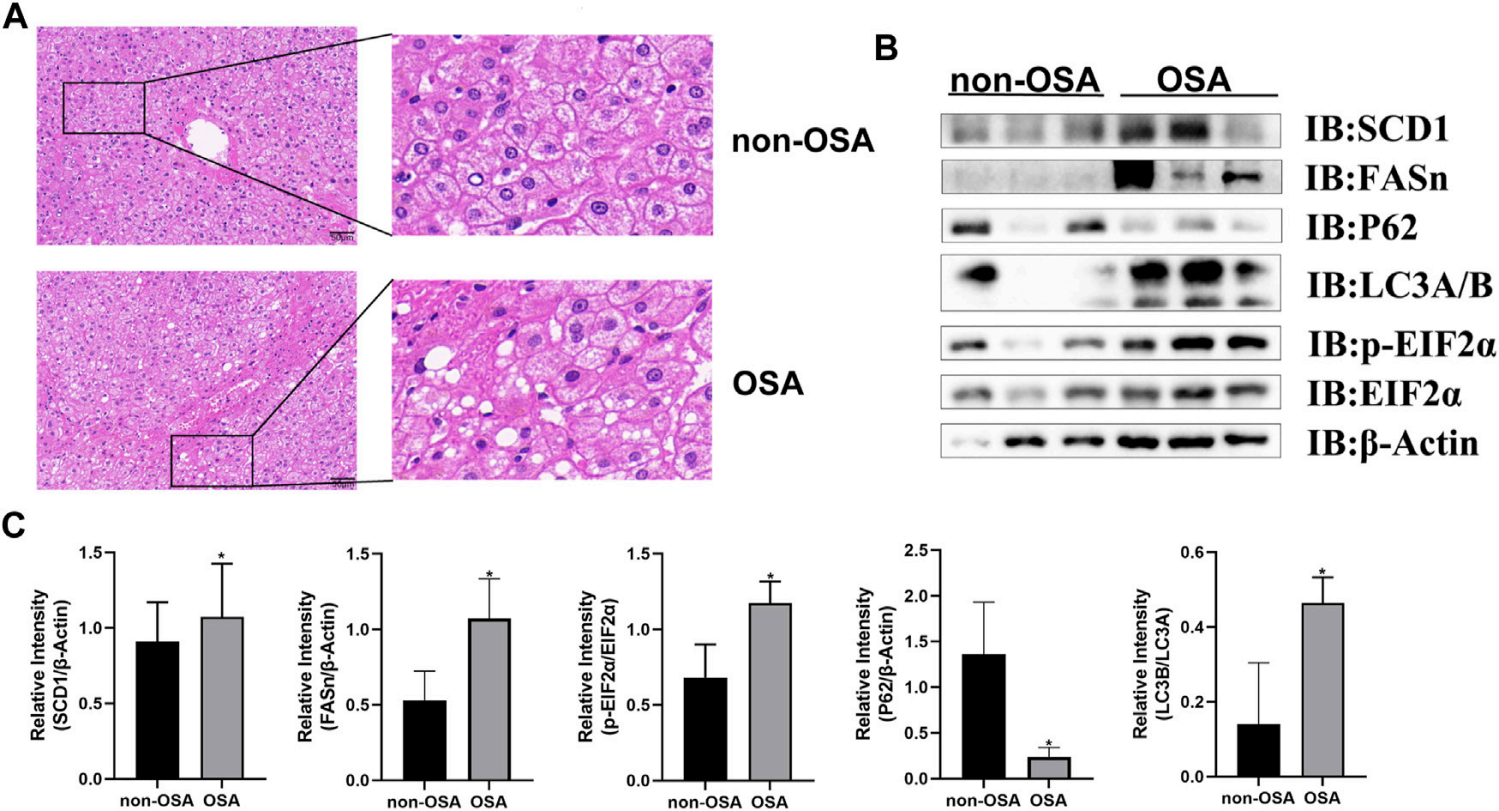
OSA patients exhibit increased hepatic steatosis and elevated levels of hepatic proteins associated with autophagy, ER stress, and lipogenesis. **(A)** H&E staining of liver tissue samples from patients with and without OSA (Scale bars: 100 and 50 μM). **(B)** Hepatic FASn, p62, LC3A/B, and p-EIF2α/EIF2α levels were measured in patients with and without OSA. **(C)** Quantification of SCD1, FASn, P62 with β-Actin and LC3B/A protein levels serving as a loading control. In addition, the p-EIF2α/EIF2α ratio was compared in individuals with and without OSA (*p < 0.05).

To explore the potential relationship between autophagic activity and ER stress in the context of OSA-associated NAFLD, samples from patients with and without OSA were analyzed to assess the incidence of hepatic steatosis. OSA patient samples revealed pronounced hepatocyte steatosis and inflammatory cell infiltration upon H&E staining that was not evident in patients without OSA (Figure 1A). NAFLD patients have previously been reported to exhibit impaired hepatic autophagic flux (Park et al., 2021). As such, autophagy-related marker proteins were detected in these patient samples to gauge autophagic flux, revealing that OSA patients exhibited an increased hepatic LC3B to LC3A ratio and lower levels of p62 as compared to non-OSA patient samples (Figures 1B,C), consistent with high levels of hepatic autophagic flux in individuals suffering from OSA. Higher levels of ER stress have been observed when autophagic activity is impaired in the development of NAFLD (Gonzalez-Rodriguez et al., 2014). Consistently, Western blotting revealed that hepatic p-EIF2α and SCD1 protein levels were elevated in OSA patients as compared to healthy controls (Figures 1B,C), suggesting that ER stress and autophagic flux are correlated in this pathogenic context. Together, these data thus indicated that dysregulated autophagic activity and ER stress are both related to the pathogenesis of OSA-associated NAFLD.

### Chronic intermittent hypoxia drives the induction of excess autophagic activity and dysregulated autophagic flux in a murine model system

**FIGURE 2:**
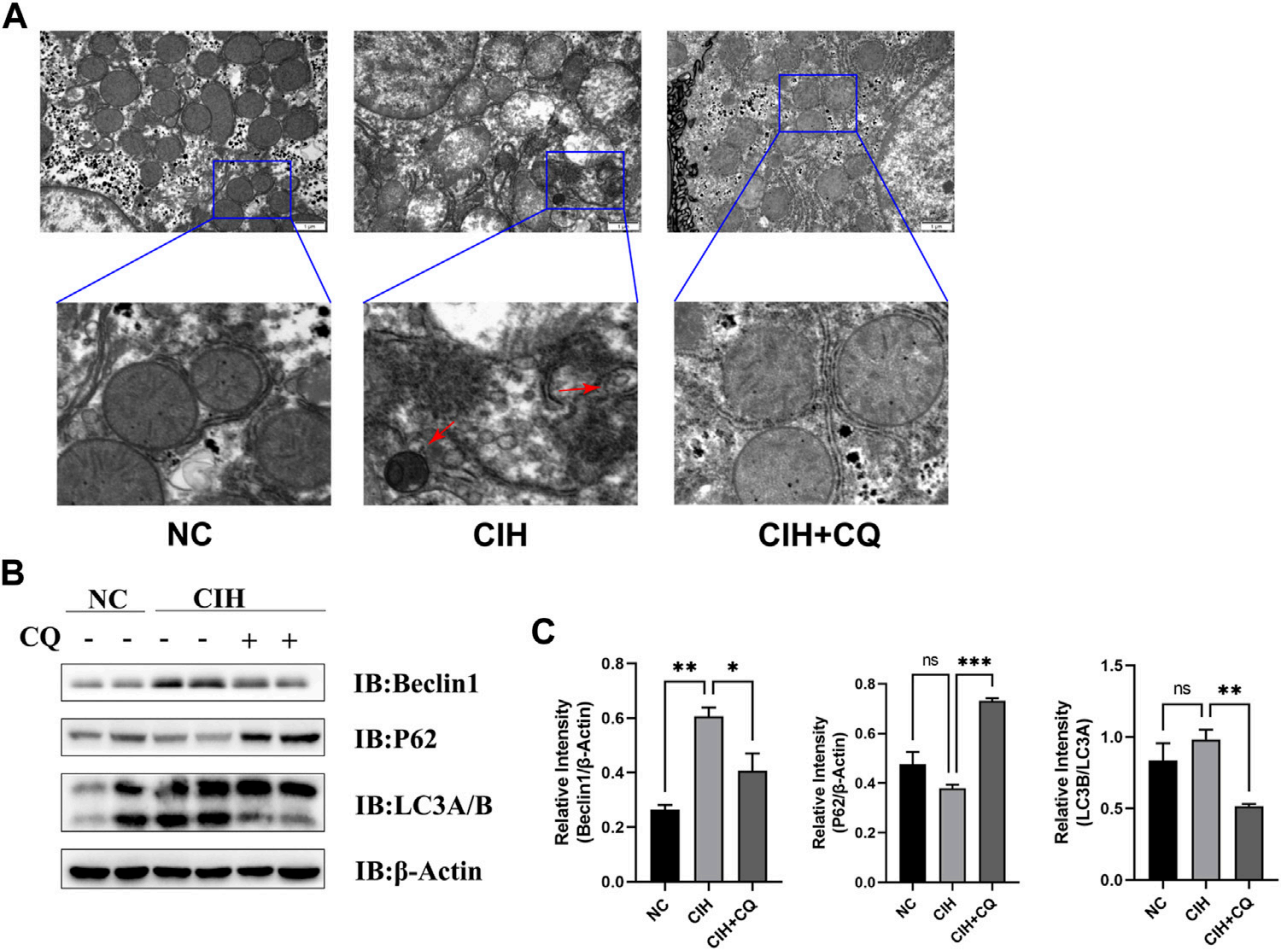
OSA model mice exhibit excess autophagic activity and autophagic flux. **(A)** CIH induced increases in autophagosome formation and accumulation in the CIH and CIH + CQ group. **(B)** Hepatic Beclin1, P62, and LC3A/B protein levels were compared among groups. **(C)** Beclin1 and P62 protein levels were normalized to β-Actin levels, and the LC3B/A ratio was assessed in different groups (Red arrow: autophagosomes; *p < 0.05, **p < 0.01, ***p < 0.001).

Next, a CIH mouse model was established in an effort to model these OSA patient phenotypes. Consistent with the changes in autophagic activity evident in hepatic samples from OSA patients, higher levels of autophagosome formation were observed in liver samples from CIH model mice (Figure 2A). Western blotting similarly revealed that these CIH model animals exhibited significantly elevated levels of the autophagosome markers Beclin1 and LC3 turnover, whereas the autophagic flux-related p62 protein was downregulated as compared to control mice. These data were indicative of excessive dysregulated autophagic activity in the hepatic tissues of these CIH-treated mice (Figure 2B). To further examine the mechanisms underlying excessive CIH-induced autophagy in the pathogenesis of hepatic steatosis, the autophagic inhibitor CQ was next used to treat these mice. The resultant increases in p62 protein level confirmed the successful blockade of autophagic activity (Figures 2B,C). These data suggested that CIH exposure can promote enhanced autophagosome formation and autophagic flux, in line with the phenotypic findings in samples from patients with OSA.

### Chloroquine administration alleviates chronic intermittent hypoxia-induced changes in hepatic metabolic activity in mice

**FIGURE 3:**
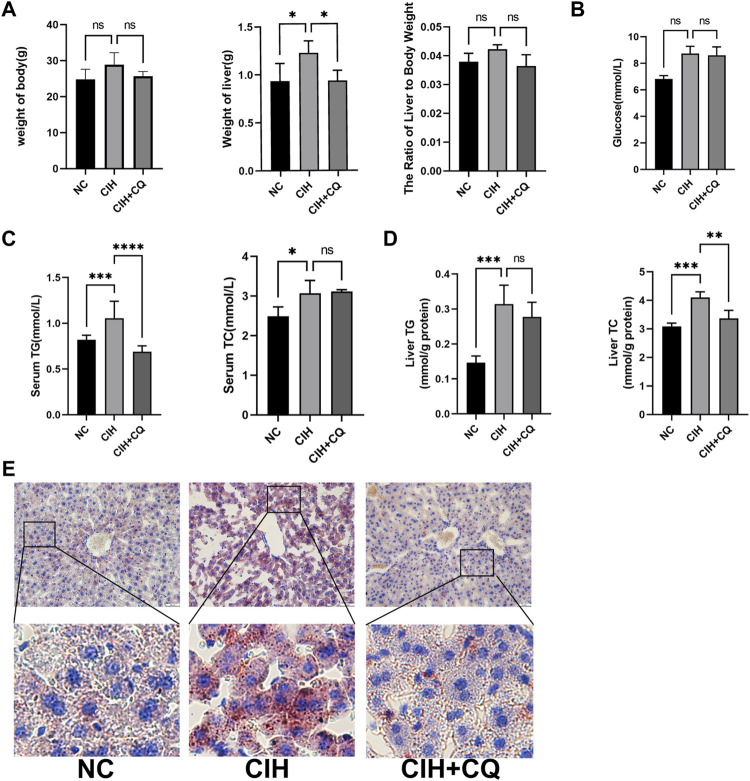
The inhibition of autophagic flux alleviates CIH-induced changes in liver indices and metabolic activity. **(A)** Body weight, liver weight, and liver weight ratio values were compared in different murine treatment groups. **(B)** Glucose levels were compared in different groups. **(C)** Serum TG and TC levels were compared in different groups. **(D)** Hepatic TG and TC levels were compared in different groups. **(E)** Oil Red O staining was used to analyze liver tissue samples from different groups (Scale bars: 100 and 50 μM) (*p < 0.05, **p < 0.01, ***p < 0.001, ns: no statistical difference).

The accumulation of lipids within the liver results in increases in liver weight. Mice in the CIH treatment group exhibited significant increases in both liver weight relative to control animals, while both of the liver weight was significant reduced in the CIH + CQ group compared with CIH group (Figure 3A).

CIH-treated mice exhibited elevated levels of blood glucose as compared to controls, while these levels were lower for mice in the CIH + CQ treatment group (Figure 3B), but these changes were not statistical significance. Serum and hepatic TG and TC levels were significantly elevated in the CIH group relative to the control group, these indicators were decreased in the CIH + CQ group (Figures 3C,D). Oil Red O staining additionally confirmed that the pronounced steatosis was evident in the livers of mice in the CIH treatment group, while such steatosis was largely reversed following CQ treatment (Figure 3E). These data thus suggest that CIH can induce excessive autophagy in the liver, thereby driving hepatic steatosis.

### Chloroquine administration suppresses the upregulation of lipogenesis-related genes in hepatocytes

**FIGURE 4:**
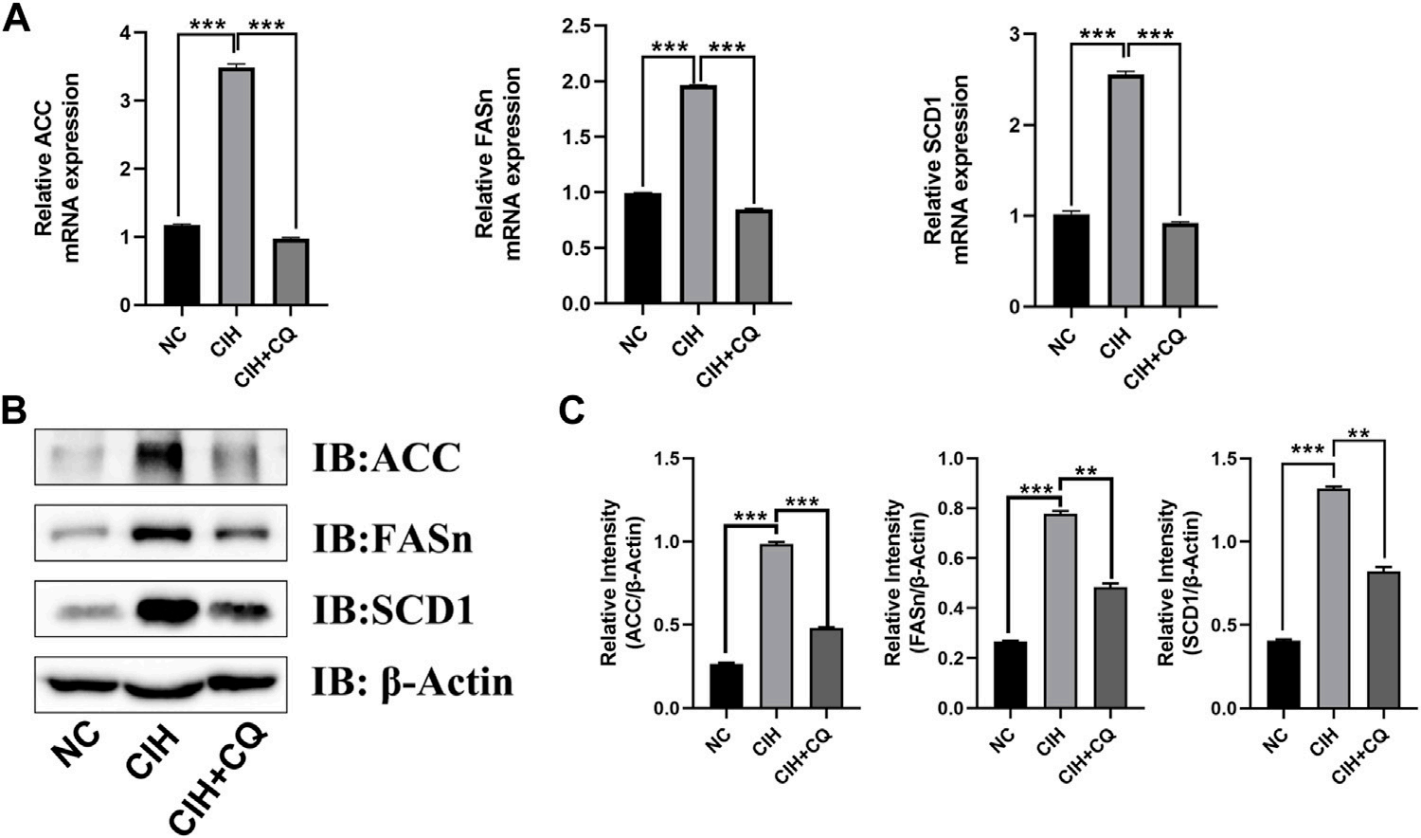
The inhibition of autophagic flux reduces hepatocyte lipogenesis-related gene expression. **(A)** Hepatic SCD1, ACC, and FASn mRNA levels were compared in different groups. **(B)** Hepatic SCD1, ACC, and FASn protein levels were compared among groups. **(C)** Quantification of ACC, FAS, and SCD1 protein levels, with β-Actin serving as a normalization control (**p < 0.01, ***p < 0.001).

**FIGURE 5:**
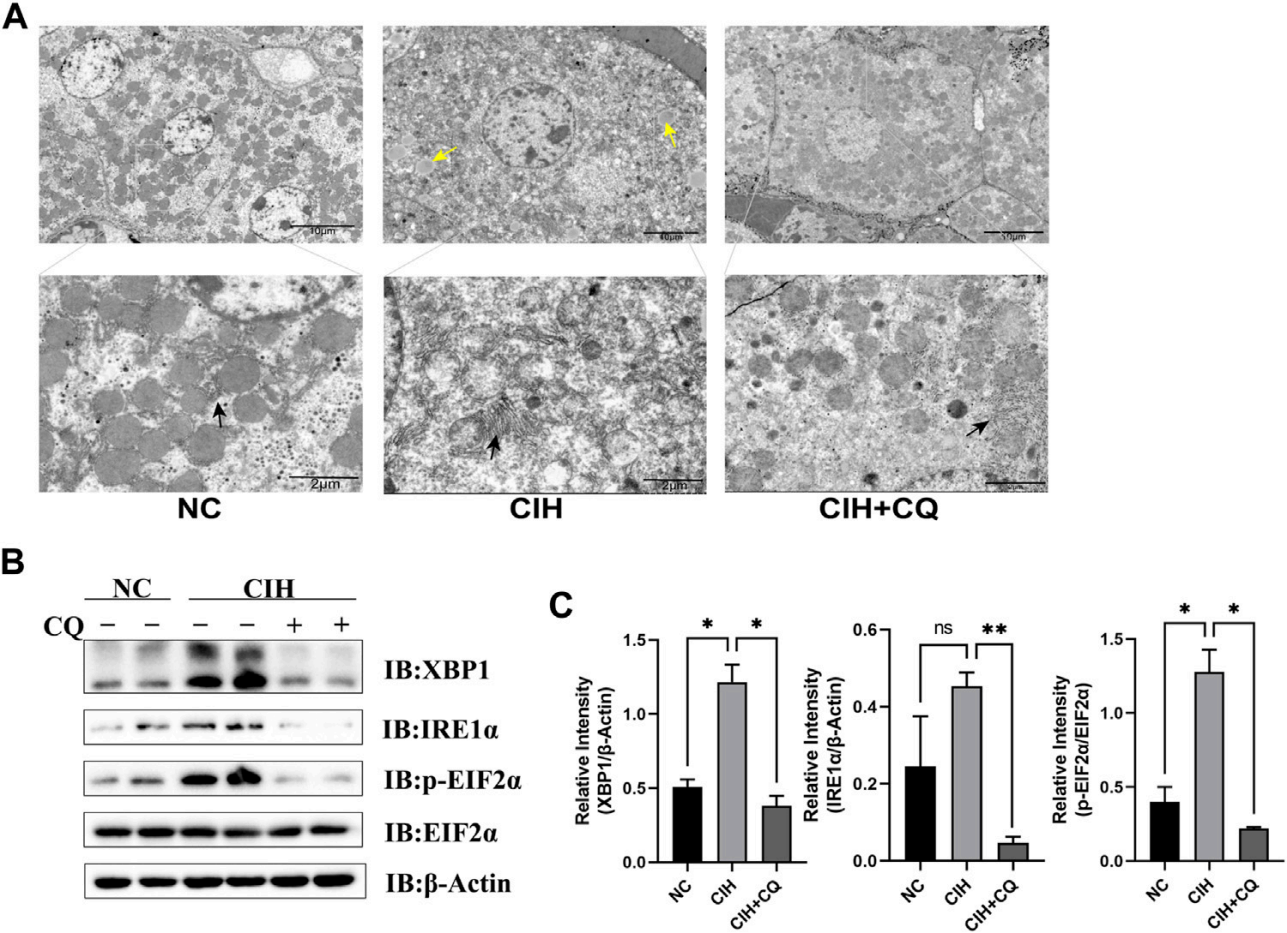
Inhibition of autophagic flux alleviated hepatocyte ER stress and LD accumulation. **(A)** Changes in ER morphology and LD accumulation in different groups were assessed. **(B)** Hepatic XBP1, IRE1α, and EIF2α protein levels were compared among groups. **(C)** XBP1 and IRE1α protein levels were normalized based on β-Actin levels, and the p-EIF2α/EIF2α ratio was compared among groups. (Yellow arrow: LDs; black arrow: ER; *p < 0.05, **p < 0.01, ns: no statistical difference).

To examine the mechanisms whereby the excessive autophagic activity induced in response to CIH can contribute to hepatic steatosis, lipogenesis-related gene expression was next assessed in liver samples from these mice. Animals in the CIH group exhibited increases in ACC, FASn, and SCD-1 mRNA levels relative to control mice (*p* < 0.01 or *p* < 0.001), while these genes were significantly downregulated following CQ administration (Figure 4A). Western blotting similarly confirmed that ACC, FASn, and SCD1 protein levels were elevated in hepatic samples from the CIH group as compared to the control group, yet were downregulated in the CIH + CQ group relative to the CIH group (Figure 4B). Hepatic steatosis is driven by LD accumulation within hepatocytes, and TEM was thus used to quantify the numbers of LDs within hepatocytes in these different treatment groups. In line with the above gene expression data, LD numbers were significantly higher in the CIH group relative to the control group, while these LD numbers were significant reduced in the CIH + CQ group relative to the CIH group. As such, CIH exposure was sufficient to induce excessive autophagic activity and LD deposition within the liver (Figure 5A), suggesting that the observed upregulation of lipogenesis-related genes in response to CIH-induced autophagy may ultimately contribute to the observed LD accumulation within murine hepatocytes.

### Chloroquine administration inhibits chronic intermittent hypoxia-driven endoplasmic reticulum stress within hepatocytes

The interplay between ER stress and autophagic activity is integral to the pathogenesis of NAFLD, while basal autophagy can protect hepatocytes against ER stress and steatosis (Gonzalez-Rodriguez et al., 2014). As such, the ability of CIH-induced excess autophagic activity to suppress ER stress in this murine model system was examined. ER enlargement was evident within hepatocytes in CIH model mice relative to mice from the control group, while CQ treatment reversed this change (Figure 5A). Samples from the CIH group additionally exhibited significantly increased XBP1, p-EIF2α levels as compared to controls, while CQ treatment reversed these changes in ER stress-related protein expression (Figures 5B,C). These results thus suggested that CIH can induce excessive autophagic activity in murine hepatocytes, in turn contributing to the development of ER stress.

### Chloroquine administration protects against hepatic inflammation in chronic intermittent hypoxia model mice

**FIGURE 6:**
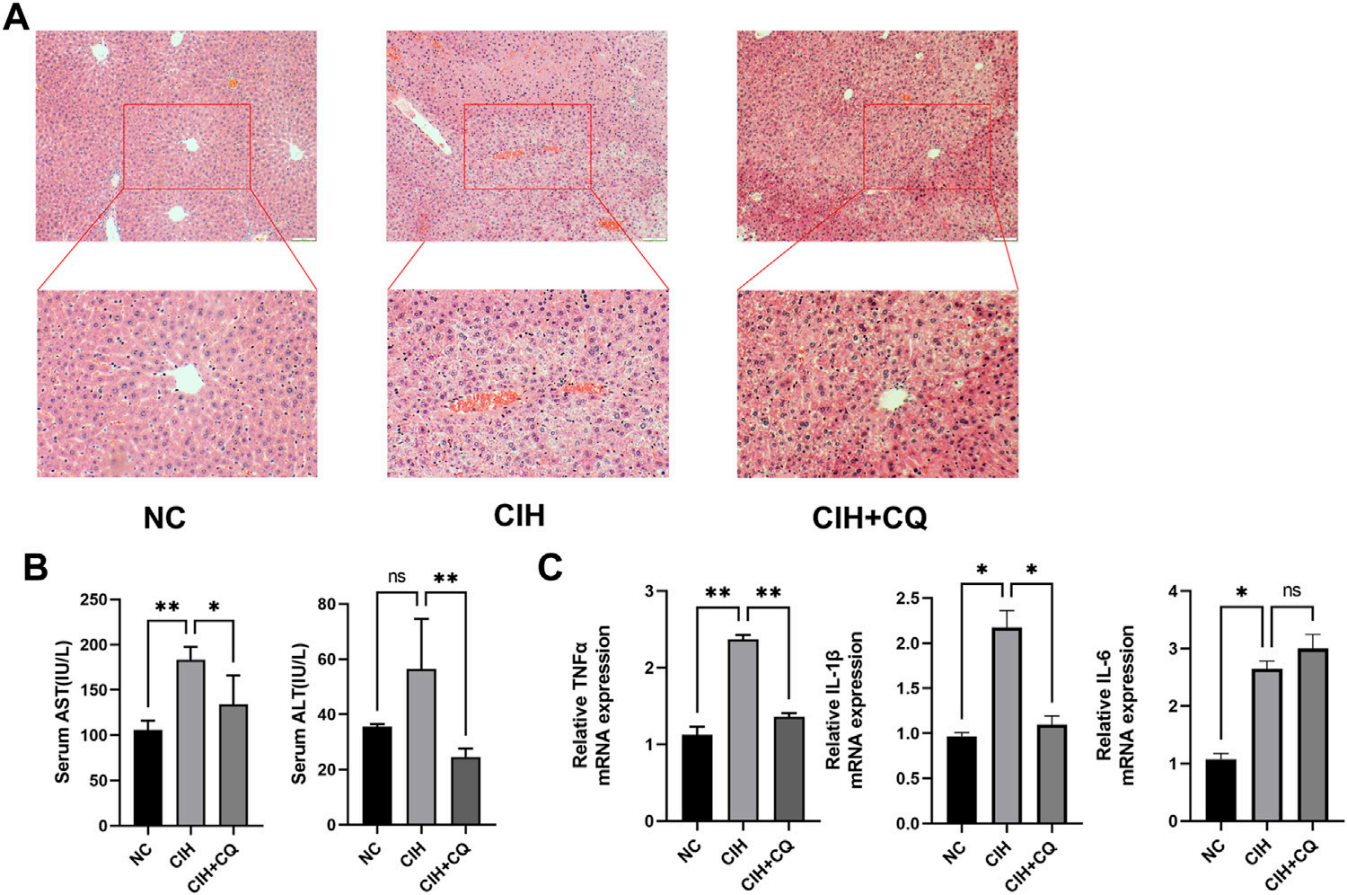
Inhibition of autophagic flux alleviates CIH-induced hepatocellular inflammation. **(A)** H&E staining of hepatic tissues from mice in the indicated groups (Scale bars: 100 and 50 μM). **(B)** Serum ALT and AST were compared among groups. **(C)** Hepatic IL-1β, IL-6, and TNF-α mRNA levels were compared among groups (*p < 0.05, **p < 0.01, ns: no statistical difference).

Inflammation can drive more rapid and severe NAFLD progression. As such, the relationship between CIH-induced autophagic activity, hepatic inflammation, and associated tissue damage was assessed. H&E staining indicated that CIH exposure was associated with higher levels of inflammatory cell infiltration and necrotic cell death, while these pathological changes were largely reversed in the CIH + CQ group (Figure 6A). Serum AST and ALT levels were higher in CIH model mice relative to controls, while these levels were significant reduced in the CIH + CQ group (Figure 6B). Consistently, hepatic tissue samples from CIH model mice exhibited higher mRNA levels for proinflammatory cytokines (TNF-α, IL-1β, IL-6) as compared to control mice, whereas CQ treatment significantly suppressed the upregulation of two of these three genes although the difference in IL-6 expression was not significant (Figure 6C). These data thus indicated that exposure to CIH can drive excessive hepatic autophagy, thus contributing to liver damage and associated inflammatory activity.

## Discussion

OSA and NAFLD are highly prevalent diseases, and there is strong clinical evidence linking OSA to the pathogenesis and exacerbation of NAFLD (Jin et al., 2018), with the association between these two conditions being independent of body weight (Tanne et al., 2005). While HIF has previously been reported to shape NAFLD progression in the context of CIH exposure (Chen et al., 2019; Mesarwi et al., 2021), the pathogenesis of NAFLD is complex and multifactorial, underscoring the need for further research. Consistently, in the present study, elevated levels of autophagic flux and ER-stress associated protein expression were observed in hepatic tissue samples from patients with OSA as compared to control samples from individuals without OSA. Lipogenesis-related gene expression was also increased in these OSA patient liver samples relative to those from healthy controls. CIH and acute intermittent hypoxia have previously been reported to differentially impact ER stress induction, with such stress being induced by the former but not the latter (Sforza and Roche, 2016). Here, a CIH mouse model was established and used to explore the pathogenesis of CIH-associated changes in hepatic lipid metabolism. Notably, the treatment of these mice with CQ resulted in the downregulation of lipogenesis- and ER stress-related genes that were induced in response to CIH exposure in these animals. CIH also drove more pronounced LD accumulation, hepatic inflammation, and steatosis, while CQ treatment reversed all of these changes, in line with prior evidence (Li et al., 2005; Zhou et al., 2021). This study is the first to our knowledge to have demonstrated that CIH can promote NAFLD development through the induction of excessive autophagic activity.

By breaking down damaged or dysfunctional organelles and proteins, autophagy can contribute to the maintenance of cellular homeostasis. Intermittent hypoxia has previously been shown to induce autophagic activity in both animal models and in clinical contexts, with one study having observed elevated Beclin-1 protein levels in OSA patient serum as compared to patients without OSA, and these Beclin-1 levels were correlated with oxygen desaturation index and apnea-hypopnea index values for these patients (Schlemmer et al., 2021). Consistently, exposure to intermittent hypoxia for 6 weeks was linked to an increase in autophagic vacuole formation and to the LC3-II/LC3-I ratio within pancreatic β-cells (Song et al., 2018). Such results are not universal, however, with one study having observed the inhibition of autophagy in a murine high-fat diet **(**HFD)/CIH model system (Ren et al., 2019). In the present study, OSA patients and CIH model mice exhibited an increase in LC3B/LC3A ratio values consistent with the induction of autophagy within hepatocytes in response to CIH exposure.

A relationship between autophagic activity and NAFLD has previously been reported pertaining to the lipophagy-mediated degradation of LDs (Martinez-Lopez and Singh, 2015; Zhao et al., 2020). The use of rapamycin and carbamazepine to induce autophagy resulted in the alleviation of insulin resistance and hepatic steatosis in a HFD-induced murine NAFLD model system (Lin et al., 2013). The specific deletion of autophagy-related genes in hepatocytes has also been found to lead to exacerbated LD deposition within these cells in animals being fed a HFD (Singh et al., 2009), thus supporting the ability of autophagy to protect the liver from the pathogenesis of steatosis. In one previous report, S100A11 overexpression was found to activate lipogenesis and autophagy, conversely resulting in increased lipid accumulation and hepatic steatosis in animals being fed a HFD (Zhang et al., 2021). FIP200 is the core mediator of autophagosome formation, and deficient hepatic FIP200 expression can result in lower liver TG levels in mice on a HFD (Ma et al., 2013). Hepatic Atg7 deletion in mice can also protect against the HFD-induced hepatic accumulation of lipids, insulin resistance, and associated obesity (Kim et al., 2013). When improperly regulated, excessive autophagic activity can lead to extensive and progressive cell death, thereby contributing to a range of pathological responses (Clarke and Puyal, 2012; Galluzzi et al., 2012).

The effects of autophagy on liver function are highly context-dependent, and can beneficially or adversely impact hepatic function. In this study, elevated levels of autophagic flux in OSA patients and CIH model mice coincided with increased hepatic FASn expression. In the CIH + CQ group, LD accumulation and hepatic steatosis were alleviated compared with CIH group. These findings thus indicated that CIH-driven hepatic autophagy plays a deleterious role in the context of the pathogenesis of NAFLD.

Basal autophagic activity is essential for cellular survival and the maintenance of homeostatic activity, but excessive or unrestrained autophagy can lead to damage. Chang et al. found that intermittent hypoxia exposure for 3 days in mice was associated with the induction of myocardial autophagy, thereby reducing myocardial cell apoptosis in response to ER stress (Chang et al., 2019). Fang et al. determined that autophagy can induce opposing effects during different stages of spinal cord ischemia-reperfusion (I/R) injury progression, with autophagic induction during the early stages of such injury being beneficial owing to the inhibition of inflammation and apoptotic cell death. Conversely, excessive, prolonged autophagic activity in this system is associated with more severe I/R injury owing to the induction of autophagic cell death (Fang et al., 2016). Under conditions of chronic stress, excess autophagy cannot effectively counteract the damaging effects of ER stress, contributing to cytotoxic cell death (Khaket et al., 2018). Yang et al. determined that Xinbao pill administration was capable of suppressing excessive autophagy and ER stress so as to protect against myocardial I/R injury (Yang et al., 2022), while Zhou et al. reported the induction of ER stress following intermittent hypoxia exposure for 4 weeks as confirmed by increased in CHOP, ATF6, and GRP78 protein levels (Zhou et al., 2014). In this study, higher autophagy-related protein levels were observed in CIH model mice after 12 weeks and in OSA patients, with a corresponding increase in ER stress-associated protein levels, while CQ administration was sufficient to suppress the ER stress induced in response to CIH. Exposure to CIH may thus lead to increases in autophagic activity and ER stress within hepatocytes, thereby resulting in adverse homeostatic cellular outcomes over extended periods of time.

The pathogenic progression of NAFLD has previously been linked to ER stress (Lebeaupin et al., 2018), in line with the results of this study, with the interplay between ER stress and autophagy being particularly important in the development of NAFLD (Flessa et al., 2021). ER stress has also been reported to mediate high glucose-induced hepatic lipid accumulation (Zhao et al., 2020). Autophagic activity can alleviate ER stress through the breakdown of excessive unfolded proteins. Baicalin, for example, can suppress ER stress via downregulating SREBP-1C and enhancing hepatic autophagic activity, thereby preventing the accumulation of lipids within the liver (Zhang et al., 2022). While studies have reported intermittent hypoxia to drive enhanced myocardial autophagy as an adaptive response to ER stress and apoptosis (Chang et al., 2019), such work was focused on the short-term outcomes associated with intermitted hypoxia rather than using a CIH model system.

Other studies have generally reported a reduction in autophagic activity in the context of NAFLD (Flessa et al., 2021). In contrast, in this study, prolonged CIH exposure was found to be associated with exacerbated liver damage characterized by histological changes and the deterioration of hepatic function, together with elevated levels of ER stress and autophagic flux in this murine model of OSA-associated NAFLD. In light of these data, we hypothesize that CIH-induced autophagic activity and consequent ER stress play an important role in the pathogenesis of NAFLD. Notably, the *in vivo* administration of CQ was sufficient to partially alleviate CIH-induced ER stress and hepatic lipid accumulation.

There are multiple limitations in this study. Firstly, the pre-operative serum TG levels were not measured in the recruited patients. Secondly, the sample of each group was small, we will increase the number of mice in further studies. Thirdly, we did not verify that CIH induced excessive autophagic activity which promoted ER stress involved in the pathogenesis of NAFLD, further research will be explored in this process.

Together, these results suggest that excessive autophagic activity induced in response to CIH can drive hepatic lipid accumulation and steatosis, with ER stress playing a central role in this pathogenic process. The novel identification of excess autophagy as a mediator of altered hepatic lipid metabolism in response to CIH highlights several promising targets that may be leveraged in an effort to treat or prevent the development of NAFLD in patients affected by OSA.

## Data Availability

The original contributions presented in the study are included in the article/supplementary material, further inquiries can be directed to the corresponding author.
